# Using the NYHA Classification as Forecasting Tool for Hospital Readmission and Mortality in Heart Failure Patients with COVID-19

**DOI:** 10.3390/jcm11051382

**Published:** 2022-03-02

**Authors:** Ioana Mihaela Citu, Cosmin Citu, Florin Gorun, Radu Neamtu, Andrei Motoc, Bogdan Burlea, Ovidiu Rosca, Felix Bratosin, Samer Hosin, Diana Manolescu, Raul Patrascu, Oana Maria Gorun

**Affiliations:** 1 Department of Internal Medicine I, “Victor Babes” University of Medicine and Pharmacy Timisoara, Eftimie Murgu Square 2, 300041 Timisoara, Romania; citu.ioana@umft.ro; 2 Department of Obstetrics and Gynecology, “Victor Babes” University of Medicine and Pharmacy Timisoara, Eftimie Murgu Square 2, 300041 Timisoara, Romania; gorun.florin@umft.ro (F.G.); radu.neamtu@umft.ro (R.N.); 3 Department of Anatomy and Embryology, “Victor Babes” University of Medicine and Pharmacy Timisoara, Eftimie Murgu Square 2, 300041 Timisoara, Romania; amotoc@umft.ro; 4 Department of Obstetrics and Gynecology, Municipal Emergency Clinical Hospital Timisoara, 1-3 Alexandru Odobescu Street, 300202 Timisoara, Romania; bogdanburlea@yahoo.com (B.B.); oanabalan@hotmail.com (O.M.G.); 5 Methodological and Infectious Diseases Research Center, Department of Infectious Diseases, “Victor Babes” University of Medicine and Pharmacy Timisoara, 300041 Timisoara, Romania; ovidiu.rosca@umft.ro (O.R.); felix.bratosin7@gmail.com (F.B.); 6 Department of Orthopedics, “Victor Babes” University of Medicine and Pharmacy Timisoara, Eftimie Murgu Square 2, 300041 Timisoara, Romania; samerhosin@gmail.com; 7 Department of Radiology, “Victor Babes” University of Medicine and Pharmacy Timisoara, Eftimie Murgu Square 2, 300041 Timisoara, Romania; dmanolescu@umft.ro; 8 Department of Functional Sciences, “Victor Babes” University of Medicine and Pharmacy Timisoara, Eftimie Murgu Square 2, 300041 Timisoara, Romania; patrascu.raul@umft.ro

**Keywords:** SARS-CoV-2, COVID-19, NYHA classification, heart failure, mortality risk, rehospitalization

## Abstract

During the COVID-19 pandemic, it was observed that patients with heart disease are more likely to be hospitalized and develop severe COVID-19. Cardiac disease takes the top position among patient comorbidities, heart failure (HF) prevalence reaching almost 5% in the general population older than 35 years in Romania. This retrospective study aimed to determine the potential use of the NYHA classification for HF in hospitalized patients with COVID-19 as prognostic tool for in-hospital mortality, length of hospitalization, and probability of rehospitalization for HF decompensation. We observed that patients with advanced HF had a history of significantly more comorbid conditions that are associated with worse disease outcomes than the rest of patients classified as NYHA I and II. However, regardless of existing diseases, NYHA III, and, especially, NYHA IV, patients were at greatest risk for mortality following SARS-CoV-2 infection. They required significantly longer durations of hospitalization, ICU admission for mechanical ventilation, and developed multiple severe complications. NYHA IV patients required a median duration of 20 days of hospitalization, and their in-hospital mortality was as high as 47.8%. Cardiac biomarkers were significantly altered in patients with SARS-CoV-2 and advanced HF. Although the study sample was small, all patients with NYHA IV who recovered from COVID-19 required a rehospitalization in the following month, and 65.2% of the patients at initial presentation died during the next six months. The most significant risk factor for mortality was the development of severe in-hospital complications (OR = 4.38), while ICU admission was the strongest predictor for rehospitalization (OR = 5.19). Our result highlights that HF patients continue to be vulnerable post SARS-CoV-2 infection. Physicians and policymakers should consider this population’s high likelihood of hospital readmissions when making discharge, hospital capacity planning, and post-discharge patient monitoring choices.

## 1. Introduction

The coronavirus disease 2019 (COVID-19) pandemic has had a substantial influence on the treatment of acute and chronic illnesses, with many disruptions to healthcare systems recorded globally as a result of several unsuccessful efforts to restrict the spread of the severe acute respiratory syndrome coronavirus 2 (SARS-CoV-2). There was an overall drop in urgent cardiovascular hospital admissions throughout the pandemic period, along with a delay in urgent treatment and an increased risk of complications. Similarly, other studies found a more than 50% decrease in heart failure (HF) hospitalizations when compared to the pre-COVID-19 timeframe.

The New York Heart Association (NYHA) classification system is a straightforward method for categorizing the severity of HF. It categorizes patients into one of four groups depending on their limitations and symptoms during physical activity; the limitations and symptoms include typical breathing difficulties, different degrees of shortness of breath, and angina discomfort. The NYHA classification has been used to evaluate the functional state of patients suffering from cardiac illness [1]. During the pandemic, patients admitted to the hospital for HF were sicker, with greater rates of NYHA class III or IV symptoms, and decompensated heart disease [2]. It is believed that these individuals avoided hospitalization during the pandemic out of fear of contracting SARS-CoV-2 infection, which ultimately deteriorated their prognosis of intrinsic heart disease [3].

Greater angiotensin-converting enzyme-2 (ACE2) receptor expression has been suggested to be a possible link between increased susceptibility to SARS-CoV-2 infection and cardiovascular disease, especially in individuals taking ACE inhibitors or angiotensin receptor blockers [4,5]. Numerous recent studies demonstrate the critical role of the ACE receptor in mediating SARS-CoV-2 infection; however, whether ACE2 expression is raised or maintained at the same level in cardiac illnesses, or as a consequence of ACE inhibitors or angiotensin receptor blockers, remains debatable. Increased circulating ACE2 levels were also reported in individuals with myocardial infarction, suggesting that this is a mechanism counteracting the renin–angiotensin–aldosterone system activation [6]. The contact between the virus and the ACE2 receptor on the host cell is the first stage in viral infection and is a critical predictor of host species range and tissue tropism. Earlier research indicated that the ACE2 receptor is highly expressed in heart tissue cells such as cardiomyocytes, cardiac myofibroblasts, and endothelial cells, and that it plays a protective function against cardiac injury [7,8].

Due to the pathophysiological processes involved in SARS-CoV-2 infection, HF is an important vulnerability during respiratory viral infections [9,10]. These pathways tend to HF decompensation and an increased risk of arrhythmia and ischemia. Additionally, medication interactions with SARS-CoV-2-targeted drugs may enhance the risk of developing cardiomyopathy, arrhythmias, and sudden death in individuals with underlying cardiovascular disease [11,12]. Secondary to the infection, the inflammatory state and cytokine production increase blood viscosity and coagulability, create endothelial dysfunction, and promote electrolyte and hemodynamic imbalance [13,14]. Numerous investigations identified HF as a significant comorbidity related with the severity of SARS-CoV-2 infection. Chronic HF as a pre-existing illness is prevalent in almost 5% of the Romanian population older than 35 [15], and was observed at a rate between 3% and 20% in hospitalized patients with SARS-CoV-2 infection, depending on the demographic investigated [16,17]. Generally, the prevalence of HF was widely reported in COVID-19 studies [18,19], but there is less information regarding the association with stages of HF, chamber implicated, and the type of dysfunction with severity of SARS-CoV-2 infection, ease of recovery after severe infection, and survival studies.

It is crucial to understand the processes behind the link between cardiac illness and increased SARS-CoV-2 susceptibility, as well as the development of severe COVID-19, in order to improve patient management and avert complications and mortality. Consequently, we designed a study aiming to investigate the potential use of NYHA classification in mortality and morbidity assessment for patients hospitalized with SARS-CoV-2 infection. The main end-point was in-hospital mortality, while the secondary target was the length of hospitalization and probability of rehospitalization for HF decompensation.

## 2. Materials and Methods

### 2.1. Study Design and Ethical Considerations

This single-center study was designed as a retrospective cohort of hospitalized patients with COVID-19 suffering from chronic HF. The setting was a tertiary hospital in Western Romania, where patients were admitted to the COVID-19 unit of the Internal Medicine Department of Timisoara Municipal Emergency Hospital during the January 2020 and January 2022 period. The research protocol was approved by the Ethics Committee of the “Victor Babes” University of Medicine and Pharmacy from Timisoara, Romania, and by the Ethics Committee of the Timisoara Municipal Hospital.

### 2.2. Inclusion Criteria and Study Variables

The inclusion criteria were set for all patients over 18 years old with a history of hospital admission for SARS-CoV-2 infection in our department, diagnosed by real time polymerase chain reaction (RT-PCR). All patients identified in the hospital database had a diagnosis of chronic HF classified by the NYHA scale. The exclusion criteria comprised incomplete patient profiles in terms of imaging studies and laboratory data, and missing patient consent in the records investigated. Data was collected by qualified doctors who participated in this study and validated the database information with existent patient paper records. The variables considered for analysis included patient background data (age, gender, and body mass index), cardiovascular risk factors (smoking, arterial hypertension, diabetes mellitus, and dyslipidemia), comorbidities at admission (cerebrovascular disease, chronic kidney disease, and chronic obstructive pulmonary disease), daily medication taken for cardiac conditions (beta-blockers, calcium channel blockers, angiotensin receptor blockers, ACE inhibitors, loop diuretics, potassium-sparing diuretics, aldosterone antagonists, antiplatelet drugs, and nitrates), vitals (pulse, temperature, systolic blood pressure, and O_2_ saturation at admission), days elapsed from first COVID-19 symptoms until hospital admission, days of hospitalization, COVID-19 severity, oxygen supplementation (no supplementation, non-invasive ventilation, or invasive ventilation), in-hospital complications, intensive-care unit admission, in-hospital mortality, overall mortality, imaging studies (cardiac ultrasound and lung computed tomography scan), and laboratory data (white blood cells, platelets, red blood cells, aspartate aminotransferase, alanine aminotransferase, total bilirubin, serum albumin, serum glucose, estimate glomerular filtration rate, D-dimers, procalcitonin, brain natriuretic peptide, creatine-kinase, myoglobin, troponin I, troponin T, and lactate dehydrogenase).

### 2.3. Statistical Analysis

The statistical analysis was performed with IBM SPSS v.26 and MedCalc v.20. We computed the absolute and relative frequencies of categorical variables. For comparison of proportions, we used Chi-square and Fisher’s tests, while for comparison of group differences in nonparametric data, the Kruskal–Wallis test was used. Quantitative and normally-distributed variables were evaluated by mean and standard deviation with the ANOVA test. The variables identified to have significant differences between comparison groups were included in a multivariate analysis adjusted for confounding factors, data being reported as odds ratio (OR), and confidence interval (CI). A Kaplan–Meyer curve was plotted for probabilities of in-hospital mortality, overall mortality, and rehospitalization for HF decompensation. The significance threshold was set for an alpha value of 0.05.

## 3. Results

The inclusion criteria were satisfied by 131 patients with HF infected with SARS-CoV-2. The baseline characteristics of the study cohort are presented in Table 1, where data was stratified by the four stages of HF corresponding to the NYHA classification. Our analysis discovered that patients’ age was significantly different in proportions between NYHA stages, where only 24.1% of patients with NYHA stage 1 were older than 65 years, compared with 73.9% in the NYHA IV group (*p*-value < 0.001). A significant difference between study groups was also observed in patients’ gender and cardiovascular risk factors. The majority of patients with NYHA stage IV were men (60.9% vs. 39.1% women, *p*-value = 0.019). Over half of the patients diagnosed with HF NYHA III and IV had important cardiovascular risk factors, the most prevalent being arterial hypertension, followed by smoking. All patients in this study had a daily prescription for HF management, where the majority followed the guidelines for decreased ejection fraction HF. There was no significant difference in treatment types between study groups, excepting the use of potassium-sparing diuretics and aldosterone antagonists (91.3% NYHA IV vs. 48.3% NYHA I, *p*-value < 0.001), respectively (73.9% NYHA IV vs. 27.6% NYHA I, *p*-value < 0.001).

Although the general characteristics of the studied population did not show too many differences by NYHA classification, as seen in Table 1, the patient presentation and outcomes were statistically significantly worse in patients with advanced HF, as described in Table 2. The proportions of patients with severe SARS-CoV-2 infection were much higher by NYHA stages III and IV (*p*-value < 0.001). These patients had a median duration from first symptoms until hospital admission of only 2 days, compared with the least affected patients with NYHA I and II that were hospitalized after a median of 7 and 6 days, respectively (*p*-value < 0.001). The length of hospital stay had a median of 20 days in patients with congestive HF, compared with a median of 10 days in patients with NYHA I HF (*p*-value < 0.001). Patients with congestive HF presented to hospital with significantly lower oxygen saturation levels and blood pressure, while they developed statistically significantly more severe in-hospital complications, as presented in Table 2.

The imaging studies performed on all patients with HF during the hospital admission for SARS-CoV-2 infection indicated that 86.9% of patients with NYHA IV had an ejection fraction lower than 30%, compared with 44.9% in the NYHA I group (*p*-value = 0.040). Other ultrasound findings were not significantly different between study groups, although the computed tomography indicated severe lung injury in patients with severe HF (*p*-value = 0.014), as seen in Table 3.

The laboratory data of the four groups of hospitalized HF patients with COVID-19, presented in Table 4, describe multiple significant differences between the four study groups. Patients with HF NYHA III and IV were observed with important alterations in their serum parameters, mostly the way the markers are regarded for congestion and cardiac damage.

The statistically significant independent risk factors for mortality and rehospitalization in patients with heart failure infected with SARS-CoV-2 is presented in Table 5. The greatest risk factor identified for mortality was the development of severe in-hospital complications (OR = 4.38, CI[3.01–5.64]), while the ICU admission was the highest risk for rehospitalization (OR = 5.19, CI[2.26–6.08].

**Figure 1:**
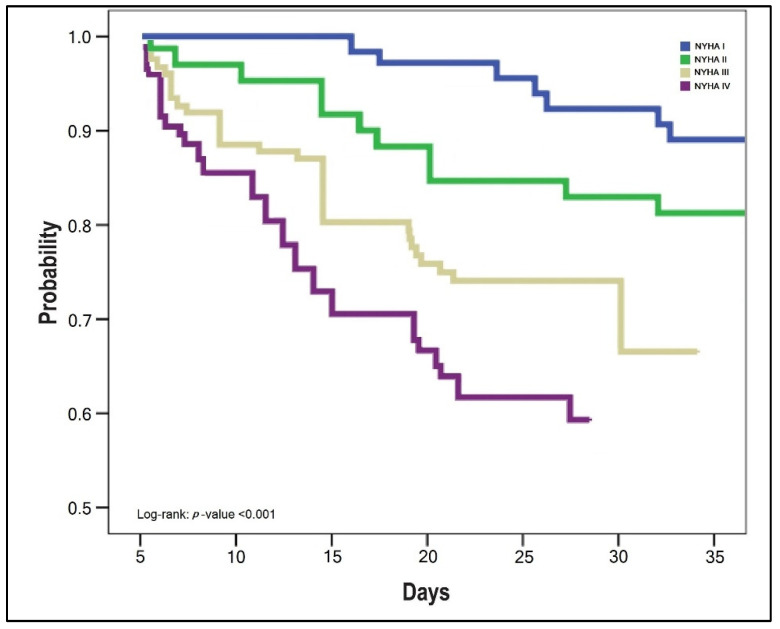
Kaplan–Meyer plot of probability for hospital readmission with cardiovascular complications after COVID-19 recovery, by NYHA classification.

**Figure 2:**
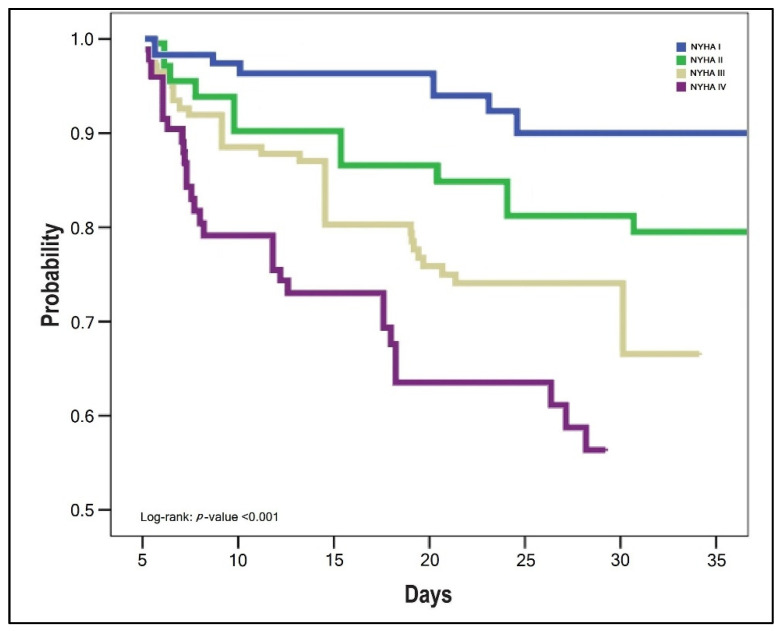
Kaplan–Meyer plot of probability for surviving the SARS-CoV-2 infection by NYHA classification.

**Figure 3:**
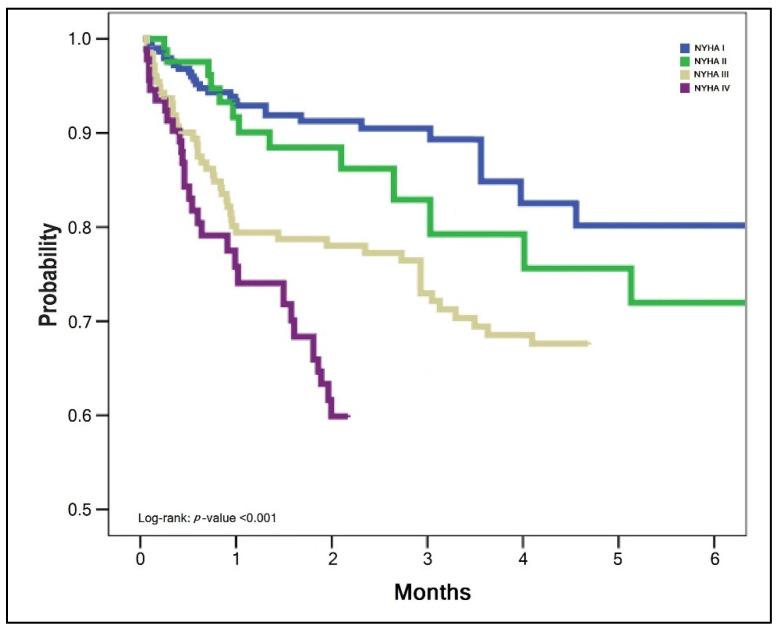
Kaplan–Meyer plot of probability for surviving after COVID-19 recovery, by NYHA classification.

The survival analysis of patients with SARS-CoV-2 infection and HF was statistically significant different between NYHA stages, as presented by the Kaplan–Meyer plots in Figure 1, Figure 2 and Figure 3. The probabilities of hospital readmission after recovering from COVID-19, the probability of surviving the SARS-CoV-2 infection during hospital admission in the acute phase, and the long-term overall mortality forecasting over 6 months were statistically significantly lower in patients with HF NYHA III and IV (Log-rank *p*-value < 0.001).

## 4. Discussion

Our research successfully determined an important estimate for negative outcomes in patients with HF infected with SARS-CoV-2. The analysis performed in the current study found a significant association between NYHA stages of HF, mortality and risk for hospital readmission. The NYHA HF classification that is based solely on patient symptomatology indicated that patients in the last stages of HF have statistically significantly higher probabilities for in-hospital mortality and overall mortality during the six months following COVID-19, as well as a significantly higher probability to be readmitted to hospital in 30 days after curing the SARS-CoV-2 infection.

Similar findings were reported in a study developed in Poland, where the authors reported a high number of deaths in patients with HF and SARS-CoV-2 infection, both in-hospital and shortly after the infection has been cleared, during a six-month time frame. The authors reported that their investigation, which was conducted on a large cohort of hospitalized patients with COVID-19, demonstrated how a history of HF impacts the in-hospital course and, more importantly, long-term follow-up. Additionally, one-third of HF patients died while hospitalized and another third died within three months after discharge [20]. As we also determined, factors such as age, gender, and comorbidities are all well-established risk factors for a severe COVID-19 course [21]. Patients with HF are often older, and their risk of complications and death after SARS-CoV-2 infection is increased by various underlying medical disorders.

Although our study followed a clinical evaluation, rather than a molecular analysis, it was previously determined that patients with a history of HF had elevated cardiac NT-proBNP and troponin I indicators and were more likely to suffer myocardial infarction [22]. Troponin elevation, on the other hand, may be detected in a wide variety of clinical situations, including HF. It should be underlined that troponin levels are mostly determined by baseline factors such as pre-existing HF or coronary artery disease. Furthermore, troponin levels were substantially greater in individuals hospitalized with COVID-19 than in those with chronic coronary syndrome [23].

Other studies evaluated the risk for rehospitalization following SARS-CoV-2 viral clearance. In a US-based study, the authors determined that by 60 days following hospital release, 27% of survivors of COVID-19 hospitalization were readmitted or died, a percentage that was lower than that of matched survivors of pneumonia or HF. However, readmission or mortality rates were significantly greater than those for pneumonia or HF in the first 10 days after COVID-19 hospitalization, indicating a time of increased risk of clinical deterioration [24]. However, the shortcomings of their research include the inability to track readmissions to other hospitals and a mostly older, male study sample, who may be at a greater risk of severe COVID-19 symptoms. In comparison, our research investigated a smaller cohort, but we collected information about patient evolution until six months post initial presentation for SARS-CoV-2 infection.

## 5. Conclusions

It is imperative to carefully evaluate patients with HF during hospitalization for SARS-CoV-2 infection, since the mortality rate, complications, and risks for hospital readmission are substantially high in advanced HF.

## Figures and Tables

**Table 1:** Comparison of general characteristics by NYHA classification of hospitalized patients with COVID-19.

Variables *	NYHA I (*n* = 29)	NYHA II (*n* = 44)	NYHA III (*n* = 35)	NYHA IV (*n* = 23)	*p*-Value
**Age, years**					<0.001
18–35	4 (13.8%)	1 (2.3%)	-	-	
35–65	18 (62.1%)	20 (45.4%)	11 (31.4%)	6 (26.1%)	
>65	7 (24.1%)	23 (52.3%)	24 (68.6%)	17 (73.9%)	
Sex					0.019
Men	10 (34.5%)	28 (63.6%)	25 (71.4%)	14 (60.9%)	
Women	19 (65.5%)	16 (36.4%)	10 (28.6%)	9 (39.1%)	
**Weight, BMI (kg/m^2^)**					0.633
<25	4 (13.8%)	4 (9.1%)	4 (11.4%)	1 (4.4%)	
25–30	7 (24.1%)	5 (13.4%)	8 (22.9%)	5 (21.7%)	
>35	18 (62.1%)	35 (79.5%)	23 (65.7%)	17 (73.9%)	
**Cardiovascular risk factors**					
Smoking	10 (34.4%)	12 (27.3%)	17 (48.6%)	14 (60.9%)	0.036
Arterial hypertension	9 (31.0%)	15 (34.1%)	24 (68.7%)	17 (73.9%)	0.003
Diabetes mellitus	8 (27.6%)	10 (22.7%)	12 (34.3%)	15 (65.2%)	0.004
Dyslipidemia	7 (24.1%)	9 (20.5%)	13 (37.1%)	13 (56.5%)	0.016
**Comorbidities at admission**					
Cerebrovascular disease	2 (6.9%)	4 (9.1%)	5 (14.3%)	7 (30.4%)	0.061
Chronic kidney disease	3 (10.3%)	3 (6.8%)	7 (20.0%)	7 (30.4%)	0.053
COPD	2 (6.9%)	2 (4.5%)	6 (17.1%)	5 (21.7%)	0.103
**Daily medication**					
Beta-blockers	22 (75.9%)	37 (84.1%)	31 (88.6%)	19 (82.6%)	0.599
Calcium channel blockers	10 (34.5%)	25 (56.8%)	19 (54.3%)	16 (69.6%)	0.078
Angiotensin receptor blockers	8 (27.6%)	12 (27.3%)	12 (34.3%)	5 (21.7%)	0.768
ACE inhibitors	21 (72.4%)	32 (72.7%)	32 (91.4%)	20 (86.9%)	0.107
Loop diuretics	23 (79.3%)	40 (90.1%)	31 (88.6%)	23 (100%)	0.115
Potassium-sparing diuretics	14 (48.3%)	16 (36.4%)	30 (85.7%)	21 (91.3%)	<0.001
Aldosterone antagonists	8 (27.6%)	12 (27.3%)	17 (48.6%)	17 (73.9%)	<0.001
Antiplatelet drugs	7 (24.1%)	17 (38.6%)	12 (34.3%)	10 (43.5%)	0.477
Nitrates	7 (24.1%)	20 (45.5%)	19 (54.3%)	8 (34.8%)	0.082

* Data reported as *n* (frequency) unless specified differently; BMI—Body Mass Index; COPD—Chronic Obstructive Pulmonary Disease; ACE—Angiotensin Converting Enzyme.

**Table 2:** Comparison of patient presentation and outcomes by NYHA classification of hospitalized patients with COVID-19.

Variables *	NYHA I (*n* = 29)	NYHA II (*n* = 44)	NYHA III (*n* = 35)	NYHA IV (*n* = 23)	*p*-Value
**COVID-19 severity**					<0.001
Mild	14 (48.3%)	10 (22.7%)	8 (22.9%)	4 (17.4%)	
Moderate	10 (34.5%)	27 (61.4%)	9 (25.7%)	4 (17.4%)	
Severe	5 (17.2%)	7 (15.9%)	18 (51.4%)	15 (65.2%)	
Days from first symptoms until admission (median, [IQR])	7 [3–11]	6 [2–9]	4 [1–7]	2 [1–4]	<0.001
Days of hospitalization (median, [IQR])	10 [7–14]	14 [9–16]	18 [8–25]	20 [10–29]	<0.001
**Vitals**					
Pulse, mean ± SD	87.1 ± 16.1	91.0 ± 16.6	95.1 ± 18.3	98.7 ± 19.0	0.083
Temperature, mean ± SD	38.4 ± 1.8	38.7 ± 1.7	38.0 ± 1.8	37.7 ± 1.2	0.089
Systolic blood pressure, mean ± SD	151 ± 21.2	136 ± 22.4	112 ± 17.6	108 ± 23.7	<0.001
**Dyastolic blood pressure, mean ± SD**	86 ± 10.8	74 ± 10.2	68 ± 9.4	66 ± 9.1	<0.001
O_2_ saturation at admission (%), mean ± SD	94 ± 4.8	92 ± 5.0	88 ± 3.9	86 ± 3.6	<0.001
**Oxygen supplementation**					<0.001
No supplementation	6 (20.7%)	6 (13.6%)	-	-	
Non-invasive ventilation	17 (58.6%)	28 (63.6%)	18 (51.4%)	11 (47.8%)	
Invasive ventilation	6 (20.7%)	10 (22.8%)	17 (48.6%)	12 (52.2%)	
**Outcomes**					
Severe in-hospital complications **	5 (17.2%)	7 (15.9%)	10 (28.6%)	11 (47.8%)	0.023
Intensive-care unit admission	6 (20.7%)	10 (22.8%)	17 (48.6%)	12 (52.2%)	0.009
In-hospital mortality	2 (6.9%)	6 (13.6%)	10 (28.6%)	11 (47.8%)	0.025
Overall mortality (6 months)	3 (10.3%)	8 (18.2%)	14 (40.0%)	15 (65.2%)	<0.001
Discharged and rehospitalized in 1 month	4/27 (14.8%)	7/38 (18.4%)	11/25 (44.0%)	12/12 (100%)	<0.001
Days from discharge until rehospitalization (median, [IQR])	24 [18–28]	21 [15–26]	16 [11–20]	10 [4–14]	<0.001

* Data reported as *n* (frequency) unless specified differently. ** Severe complications include: myocardial infarction, myocarditis, malignant arrythmia, pulmonary embolism, and acute pulmonary edema; IQR—Interquartile Range.

**Table 3:** Comparison of imaging studies by NYHA classification of hospitalized patients with COVID-19.

Variables *	NYHA I (*n* = 29)	NYHA II (*n* = 44)	NYHA III (*n* = 35)	NYHA IV (*n* = 23)	*p*-Value
**Cardiac ultrasound**					
Left ventricle ejection fraction					0.040
≥40%	7 (24.1%)	8 (18.2%)	5 (14.3%)	2 (8.7%)	
40–30%	9 (31.0%)	11 (25.0%)	4 (11.4%)	1 (4.4%)	
<30%	13 (44.9%)	25 (56.8%)	26 (72.3%)	20 (86.9%)	
Atrial fibrillation/flutter	8 (27.6%)	10 (22.7%)	9 (25.7%)	9 (39.1%)	0.547
Cardiac thrombus	2 (6.9%)	3 (6.8%)	3 (8.6%)	2 (8.7%)	0.986
Mitral valve stenosis	-	3 (6.8%)	4 (11.4%)	2 (8.7%)	0.361
Mitral valve regurgitation	4 (13.8%)	5 (11.4%)	3 (8.6%)	3 (13.0%)	0.918
Aortic valve stenosis	1 (3.4%)	1 (2.3%)	1 (2.9%)	-	0.858
Aortic valve regurgitation	2 (6.9%)	3 (6.8%)	1 (2.9%)	2 (8.7%)	0.744
Segmental wall motion abnormality	4 (13.8%)	4 (9.1%)	3 (8.6%)	5 (21.7%)	0.415
Pericardial effusion	2 (6.9%)	3 (6.8%)	2 (5.7%)	2 (8.7%)	0.978
Right ventricular dysfunction	3 (10.3%)	3 (6.8%)	1 (2.9%)	2 (8.7%)	0.671
High pulmonary artery pressure **	-	2 (4.5%)	1 (2.9%)	1 (4.4%)	0.710
**Lung injury on CT scan**					0.014
Mild (˂30%)	14 (48.3%)	17 (38.6%)	11 (31.4%)	3 (13.0%)	
Moderate (30–60%)	8 (27.6%)	19 (43.2%)	9 (25.7%)	7 (30.4%)	
Severe (>60%)	7 (24.1%)	8 (18.2%)	15 (42.9%)	13 (56.5%)	

* Data reported as *n* (frequency) unless specified differently; CT—Computed Tomography; ** Higher than 25 mmHg.

**Table 4:** Comparison of laboratory profile by NYHA classification of hospitalized patients with COVID-19.

Variables *	Normal Range	NYHA I (*n* = 29)	NYHA II (*n* = 44)	NYHA III (*n* = 35)	NYHA IV (*n* = 23)	*p*-Value
WBC (thousands/mm^3^)	4.5–11.0	13.2 ± 4.1	12.6 ± 4.5	12.8 ± 3.9	11.4 ± 5.8	0.535
Platelets (thousands/mm^3^)	150–450	308 ± 28	284 ± 31	206 ± 23	127 ± 44	0.009
RBC (millions/mm^3^)	4.35–5.65	4.41 ± 0.8	4.28 ± 0.9	4.06 ± 1.1	3.81 ± 1.8	0.235
AST (U/L)	10–40	25 ± 4.2	27 ± 5.4	31 ± 5.7	36 ± 6.1	<0.001
ALT (U/L)	7–35	21 ± 3.3	22 ± 3.1	24 ± 3.8	25 ± 4.0	0.001
Total bilirubin (g/dL)	0.3–1.2	0.7 ± 0.1	0.8 ± 0.3	0.8 ± 0.2	0.9 ± 0.5	0.119
Serum albumin (g/dL)	3.4–5.4	4.1 ± 0.5	4.0 ± 0.3	3.2 ± 0.3	2.6 ± 0.6	<0.001
Serum glucose (mmol/L)	60–125	92 ± 10.8	93 ± 10.3	96 ± 9.5	101 ± 11.4	0.009
eGFR (mL/min/1.73 m^2^)	>60	73.2 ± 5.4	70.4 ± 6.1	62.8 ± 5.9	56.9 ± 7.3	<0.001
D-Dimers (µg/mL)	<0.5	1.3 ± 0.4	2.2 ± 0.6	3.7 ± 1.0	7.4 ± 2.1	<0.001
Procalcitionin (µg/L)	<0.1	0.2 ± 0.1	0.4 ± 0.1	0.8 ± 0.2	1.3 ± 0.2	<0.001
BNP (pg/mL)	<100	241 ± 47	308 ± 66	559 ± 84	1572 ± 146	<0.001
CK-MB (U/L)	5–25	22 ± 4.1	24 ± 4.7	29 ± 4.9	38 ± 6.2	<0.001
Myoglobin (nmol/L)	1.2–3.6	1.4 ± 0.2	1.9 ± 0.3	3.1 ± 0.6	4.7 ± 0.9	<0.001
Troponin I (ng/mL)	0–0.4	0.1 ± 0.1	0.2 ± 0.1	0.3 ± 0.1	0.6 ± 0.2	<0.001
Troponin T (ng/mL)	<14	11 ± 1.3	14 ± 1.5	22 ± 1.9	25 ± 2.7	<0.001
LDH (U/L)	140–280	204 ± 28	261 ± 33	288 ± 37	341 ± 41	<0.001

* Data reported as mean ± SD unless specified differently; WBC—White Blood Cells; RBC—Red Blood Cells; AST—Aspartate Aminotransferase; ALT—Alanine Aminotransferase; eGFR—Estimated Glomerular Filtration Rate; BNP—Brain Natriuretic Peptide; CK-MB—Creatine Kinase-Myoglobin Binding; LDH—Lactate Dehydrogenase.

**Table 5:** Multivariate risk factor analysis for mortality and rehospitalization.

Factors	Mortality OR (95% CI)	*p*-Value	Rehospitalization OR (95% CI)	*p*-Value
Age (>65)	2.07 (1.44–3.01)	0.008	2.64 (1.82–3.97)	0.004
Cardiovascular risk factors (>2)	2.35 (2.01–2.94)	0.002	3.09 (1.96–5.44)	0.001
Severe COVID-19	2.92 (1.26–4.05)	0.001	3.35 (2.14–5.83)	<0.001
Invasive ventilation	3.04 (1.59–4.71)	<0.001	3.88 (2.43–6.08)	<0.001
Severe in-hospital complications	4.38 (3.01–5.64)	<0.001	4.92 (2.58–6.67)	<0.001
ICU admission	3.42 (2.01–4.66)	<0.001	5.19 (2.26–6.08)	<0.001
Left ventricle ejection fraction (<30%)	1.89 (1.16–3.07)	0.036	3.07 (2.19–4.86)	0.002

OR—Odds Ratio; CI—Confidence Interval.

## Data Availability

The data presented in this study are available on request from the corresponding author.
